# A Fistula Knife

**Published:** 1882-11

**Authors:** 


### A Fistula Knife.

The instrument in use here seems worthy of notice. It consists, simply, of an ordinary curved bistoury, mounted on a fixed handle, the point of the bistoury being prolonged in the form of a probe to the length of three or more inches. The method of using it is, the patient being in position, the left index finger, oiled, is inserted in the bowel, the probe end of the instrument passed into the sinus, the internal opening found, and the point of the instrument carried outside of the sphincter. To complete the operation, the knife is advanced and the band severed. The advantage being, that with one instrument, and at a single movement, the operation is done.—*The St. Louis Med. and Surg. Jour.*

